# Cathodic Corrosion: A Quick, Clean, and Versatile Method for the Synthesis of Metallic Nanoparticles**

**DOI:** 10.1002/anie.201100471

**Published:** 2011-05-27

**Authors:** Alexei I Yanson, Paramaconi Rodriguez, Nuria Garcia-Araez, Rik V Mom, Frans D Tichelaar, Marc T M Koper

**Affiliations:** Leiden Institute of Chemistry, Leiden UniversityPostbus 9502, 2300RA Leiden (The Netherlands); Ultrafast Spectroscopy group, FOM Institute for Atomic and Molecular Physics (AMOLF)Postbus 41883, 1009DB Amsterdam (The Netherlands); Kavli Institute of NanoScience, Delft University of TechnologyLorentzweg 1, 2628 CJ Delft (The Netherlands)

**Keywords:** electrochemistry, metal anions, nanoparticles, platinum, transition metals

In many important chemical reactions metals are used as catalysts. As only the surface of these, often very expensive, catalysts is involved in the reaction, it is beneficial to maximize the surface-to-volume ratio by decomposing the metal in small nanoparticles. Mechanical means have proven inadequate, and therefore the development of methods for chemical synthesis of metal nanoparticles has been an extremely active area of research for the last 30 years. Remarkable advances have been achieved in the preparation of highly dispersed, homogeneously small nanoparticles by the development of less complicated procedures.[1–8] At the basis of all known synthetic methods lies the reduction reaction of metal cations. To limit the number of reduced cations per nanoparticle and keep its size as constant as possible, extra chemical stabilizers are invariably added.[1–7] Stabilizers contaminate the final product and adversely affect its performance, for example, in catalysis[9–13] and biological applications.[14, 15] Here, we report a radically different, simple, and counterintuitive method of nanoparticle synthesis, opening a new direction towards producing nanomaterials with improved functional properties. The method is based on extreme cathodic polarization of a metal, leading to the formation of cation-stabilized metal anions, which then act as precursors to the formation of nanoparticles.

We will use this method on the example of platinum, a metal with one of the highest oxidation potentials. This property makes platinum very difficult to etch, and therefore attractive for applications for which corrosion must be excluded. In the past, enhanced etching of Pt and its alloys has been achieved by applying high alternating current (ac) voltages.[16] The formation of some black species had been observed,[17, 18] which were attributed to platinum chloride. This was not surprising: platinum oxidizes at +1.188 V[19] and readily forms complexes with chloride anions. The surprising observation is that this black platinum product is produced at negative potentials, at which platinum, as any other metal, is cathodically protected from oxidation. Originally reported more than 100 years ago by Fritz Haber,[20, 21] this effect had been studied by Soviet electrochemists in the past.[22] Here, for the first time we show that under these seemingly unlikely conditions metal nanoparticles form on the surface of an electrode if we use direct current (dc) potentials, and they come off the surface if we apply an ac voltage. We discuss the mechanism and the generality of this extremely simple method for the synthesis of nanoparticles and show the superior properties of these Pt nanoparticles relative to those of commercial catalysts.

We performed the following simple experiment to demonstrate cathodic corrosion of platinum. Using a standard three-electrode electrochemical cell controlled by a potentiostat we recorded a cyclic voltammogram (CV) of a platinum wire in sulfuric acid solution (Figure 1 a, gray curve). As is well-known, the region between 0.05 and 0.4 V of the CV is assigned to hydrogen adsorption/desorption on platinum, and this blank experiment is used to determine the electroactive area of the electrode.[23] Next, we subjected the same electrode to a cathodic treatment in an alkaline solution. We observed a visible change of the surface: the submerged part of the shiny platinum wire became dull black. Subsequently, we recorded another CV (Figure 1 a, black curve), which shows a striking difference to the initial CV. Apparently, the area of our electrode had increased more than tenfold under conditions where it should have been protected from any changes! This drastic change of the surface of the Pt electrode is visualized in scanning electron microscopic images. Upon magnification we see that the whole surface of the wire has become an agglomeration of nanoparticles (Figure 1 b,c). These nanoparticles are rather uniform in size and seem to be well-attached to the host wire. They are so well-attached that even vigorous evolution of H_2_ gas during the cathodic treatment was not able to remove them.

**Figure 1:**
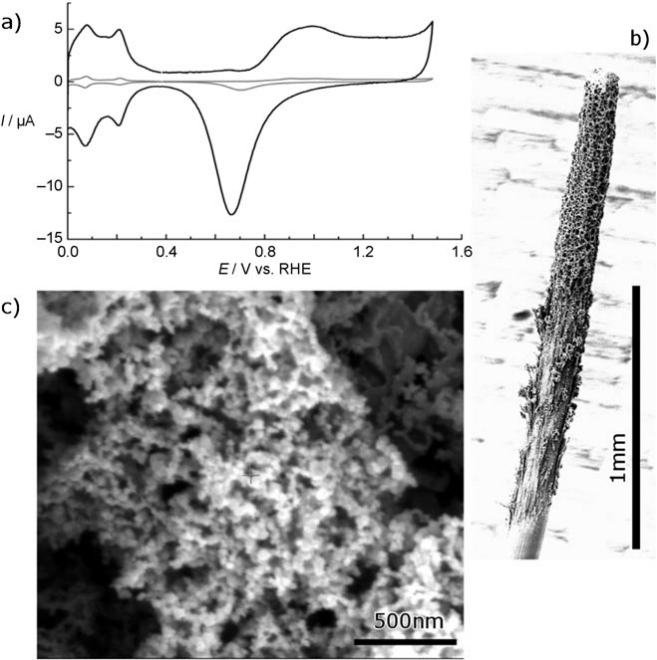
a) Cyclic voltammogram of a Pt wire 135 μm in diameter, submerged by 1 mm in 0.5 m H_2_SO_4_ before (gray) and after (black) the wire was held for 1000 s at a dc of −10 V (−7.2 V vs. HgO) in 10 M NaOH. Graphite is used as anode to rule out the formation of interfering species by anodic dissolution. Sweep rate: 50 mV s^−1^. b,c) Typical scanning electron microscopic images of a well-rinsed Pt electrode after cathodic treatment.

The spongy platinum prepared by this method is of limited practical use. We need nanoparticles dispersed in solution or transferred to a supporting electrode. By simply applying a nonoxidizing ac voltage to a platinum electrode in the same solution we decomposed the whole bulk wire in minutes, while the solution turned into a black suspension of nanoparticles.[24]

To understand these observations, we outline the most important experimental findings. Cathodic treatment of a metal electrode in an alkaline solution at extreme negative potentials causes corrosion and formation of the nanoparticles at the surface of the electrode. Little or no change to the surface of the electrode is observed if the electrolyte is an acid, that is, it contains only protons; adding an alkali salt triggers the process of corrosion. This salt has not necessarily to be composed of metallic cations; both ammonium and tetraalkylammonium salts also facilitate corrosion. Scanning electron microscopic images of the surface not obstructed by nanoparticles reveal crystallographic etching patterns.[24]

Extreme negative potentials cause vigorous reduction of protons and/or water molecules at the electrode. This process creates an extremely high pH at the interface of the solution and the electrodeeven in low-pH electrolytes, and possibly aids in the production of short-lived reactive intermediates. The dispersion is fast in concentrated alkaline solutions, and especially, in molten NaOH.[24]

A metal electrode in an alkaline solution rapidly decomposes into a suspension of metallic nanoparticles when subject to an ac voltage of a few volts from peak to peak. Going to more cathodic values yields shorter times for the decomposition. The process continues if the ac is offset so that the metal is cathodic during the whole ac cycle.

The method works well in concentrated solutions of sulfates, nitrates,[25] and chlorides[16] of alkali and alkaline earth salts, and even in tetraalkylammonium hydroxides.[24] Every metal we have examined so far has shown decomposition under ac bias.[*]

The first observation excludes corrosion through the formation of metal hydrides as well as alkali metal reduction and alloying with subsequent leaching; this is in stark contrast with the existing literature.[22] The scanning electron microscopy (SEM) results clearly indicate that we observe a chemical process of metal dissolution that causes etch pits on the surface, rather than physical disintegration.[18] The second observation, when the electrode is at a high cathodic potential, indicates a strong nonequilibrium situation in which little or no free water is available. Water is being reduced and the generated OH^−^ anions are stabilized by the remaining cations, that is, Na^+^ cations. The solution becomes locally very aprotic and resembles molten NaOH close to the interface of the solution and the platinum electrode. According to the third observation, we are dissolving platinum into this water-free layer, but this ionic platinum intermediate is short-lived and is quickly converted back to its metallic state and agglomerates in the form of nanoparticles. Commonly, metals dissolve as cations through an oxidative process. An oxidation reaction would be inhibited by going to more cathodic potentials, while our process benefits from more cathodic potentials. Moreover, according to the fourth observation, a whole range of anions, not only hydroxide ions, produce the same effect.

At this point it is opportune to comment on related observations of changes in the structure of platinum electrodes under repeated potential cycling.[26] Sun and co-workers have used a similar method for dissolution and re-deposition of platinum nanoparticles, which led to high-index nanoparticles with very interesting catalytic properties.[27] We stress that in these methods, anodic dissolution is considered to be the driving force for the structural changes. In our method, cathodic dissolution of the nanoparticles stabilized by cations other than protons, is the key to explaining the observed structural changes. The presence of cations is necessary for the cathodic dissolution and also rules out any physical destruction effect such as glow discharge electrolysis.[24]

All the above points lead to the conclusion that during cathodic corrosion metal anions rather than cations form, and hence the corrosion proceeds through reduction rather than oxidation. The essential presence of (alkali) cations which stabilize the metal anion corroborates our hypothesis further. These types of metal-alkali compounds, called Zintl phases, have been described, although under different experimental conditions,[22, 28–31] but all reports have in common that the studied metal anion was intolerant to even traces of moisture, because water readily oxidizes the metal anion. Here, we present compelling evidence for the existence of cation-stabilized metal anions as precursors to the formation of nanoparticles in aqueous solutions, implying an entirely new chemical route for the synthesis of nanoparticles. Unfortunately, because of concomitant vigorous hydrogen evolution at the cathode, the interface is not directly accessible to spectroscopic studies. The poor stability and short lifetime of the anion complex in aqueous solution further complicates its direct detection. The stability of bare monoatomic metal anions in water has been estimated theoretically.[32]

Based on these observations, the following mechanism is proposed for the decomposition of a metal electrode in metallic nanoparticles at a high cathodic potential:

- a water-free layer of high pH is created at the solution–metal interface,
- the metal is reduced to its anionic form and stabilized by the cations in this layer,
- upon encountering free water molecules and other oxidative species the anionic metal intermediate is reoxidized to the metallic state, and
- these metal atoms agglomerate and form nanoparticles.

Since the lifetime of these anions is very limited and the water-free region is extremely dynamic because of the vigorous hydrogen evolution, the oxidation of the anions and the formation of nanoparticles occurs in close proximity to the cathode,[24] so that in most cases the nanoparticles are attached to the surface of the cathode. This generic mechanism is not very sensitive to the initial composition and the pH of the electrolyte, as long as stabilizing cations are present,[24] and works for other metals as well.

We have confirmed the formation of suspended nanoparticles in ac experiments for Pt, Au, Cu, Ag, Ni, Rh,[24], and also for Si, Nb, and Ru. Transmission electron microscopic images of platinum and gold nanoparticles, shown in Figure 2, help us to visualize what Haber described as “metal dust”.[21] The size of the nanoparticles produced by cathodic decompostion varies from 3 to 30 nm as shown in the collection of images recorded by transmission electron microscopy (TEM). At higher magnification the lattice spacing of metallic Pt and Au is observed, respectively (Figure 2). At present no effort has been made to control either the size or preferential crystallographic orientation of the nanoparticles. However, it is conceivable that by varying such parameters as ac voltage, waveform, frequency, dc offset, temperature, stabilizing cation, metal pretreatment, concentration, and additives in the solution we can synthesize nanoparticles with predefined properties.

**Figure 2:**
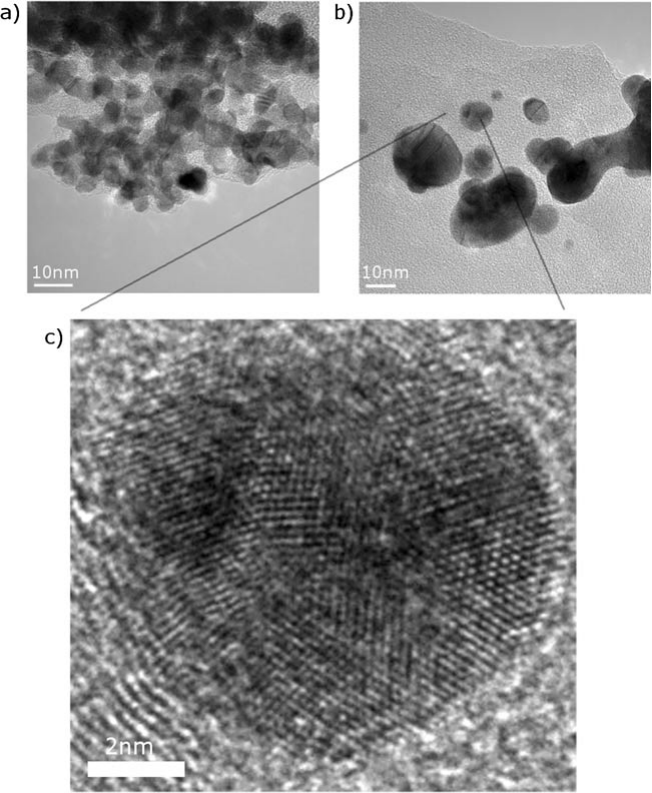
TEM images of a) platinum and b,c) gold nanoparticles extracted from solution. The size varies between 3 and 30 nm in diameter, and Au is much more prone to agglomeration. The lattice spacing as well as the energy dispersive X-ray spectra measured on all samples correspond exactly to platinum and gold, respectively.[24]

To assess if the platinum nanoparticles produced by cathodic corrosion possess useful catalytic properties, we performed a series of electrochemical experiments to test their ability to oxidize carbon monoxide and methanol (Figure 3). The latter is particularly attractive because methanol is compatible with the existing infrastructure for fuels and has a high energy density. The main problem of the oxidation of methanol on platinum is the formation of poisoning species (CO) as intermediates which are difficult to oxidize.[33, 34] Therefore, it is desirable to produce platinum nanoparticles with high catalytic activity for the oxidation of both methanol and CO. In Figure 3 the activity of the Pt nanoparticles produced by cathodic corrosion is compared to that of a state-of-the-art commercial TKK sample.[35] Three important observations are made. First, oxidation of CO takes place on our nanoparticles at an overpotential that is approximately 0.1 V lower than on the TKK sample. Second, the area under the stripping peak of CO, corresponding to the surface area of Pt onto which CO was adsorbed, is about two times lower for our nanoparticles than for the commerical TKK sample. This indicates that our nanoparticles are around 1.5 times larger on average than the commercial nanoparticles, and have a lower effective surface area per gram of platinum. Nevertheless, the intrinsic catalytic activity of our nanoparticles overcompensates for this effect, that is, for the oxidation of methanol the maximum current per gram of our nanoparticles is twice that of the TKK nanoparticles. The CVs obtained in the blank experiments on the platinum electrode (Figures 1 a and 3 a) as well as the ex situ characterization[24] show that the cathodic treatment yields clean high-surface-area Pt. This inherent cleanliness of the cathodic nanoparticles as well as the increased density of low-coordinated surface sites are likely to facilitate the catalytic enhancement. Although pure platinum is not the catalyst of choice for the oxidation of methanol, this simple example shows that our nanoparticles, produced without further purification by cathodic corrosion and prior to any optimization, show a superior catalytic activity over a state-of-the-art commercial Pt catalyst. Ongoing experiments for the optimization of the activity and selectivity of the catalysts produced by this method show encouraging results.

**Figure 3:**
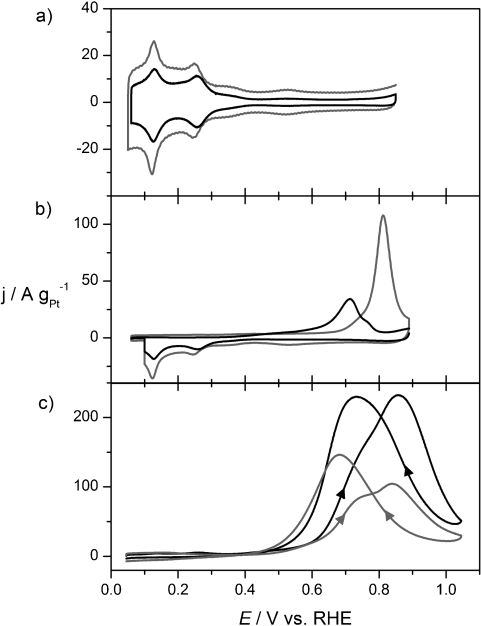
The electrocatalytic properties of platinum nanoparticles synthesized by cathodic corrosion (black) and commercial ones (gray).[35] For a proper comparison, the potential is plotted versus the current density per gram of Pt. a) Hydrogen adsorption, b) stripping of a CO monolayer, and c) oxidation of methanol. Electrolyte: 0.5 M H_2_SO_4_. Scan rate: 50 mV s^−1^. The nanoparticles are synthesized by a 10 V peak-to-peak square waveform signal in 1 M NaOH.

Finally, the simplicity and versatility of this synthetic method should be emphasized. A metal wire and an electrolyte solution are only used as reagents, and the produced nanoparticles are clean and ready to use. This method is advantageous to many conventional methods[1, 10] that require an additional purification step to clean the nanoparticles from organic reagents used in the synthesis,[11] saves time, and greatly increases the accessibility of these nanoparticles to scientists from other disciplines.

In summary, we have discovered that cathodic corrosion provides a new way to produce nanoparticles of a wide variety of metals by a hitherto entirely unexplored solution-phase chemistry. Based on our extensive experiments and observations we conclude that the reaction must proceed through the formation of cation-stabilized metal anions. The method is simple, efficient, robust, and cheap, may have many direct applications both in fundamental and applied research (biomedicine, optics, and electronics), and creates small, clean, and active nanoparticles directly usable as catalysts. Further experiments that aim at tailoring the size, shape, and composition of these nanoparticles for the purpose of tuning their catalytic activity and selectivity are under way.[36]

## Experimental Section

To obtain clean and reproducible conditions, prior to each experiment the cell and all glassware were immersed overnight in an acidic solution of KMnO_4_. Next, the solution was removed and the residual MnO_4_^−^ anions were washed out with an acidic solution of H_2_O_2_ and sulfuric acid in a 3:1 ratio. Finally, the cell and all glassware were thoroughly washed several times with boiling ultrapure water (Millipore MilliQ Gradient A10 system, 18.2 MΩ cm, 3 ppb of the total organic carbon).

The experiments were carried out in aqueous solutions prepared from high-purity NaOH (99.995 %), LiOH (99.995 %), KOH (99.99 %), CsOH (99.95 %), Na_2_SO_4_ (99.99 % trace metal basis), NaCl (99.5 %, BioXtra), (NH_4_)_2_SO_4_ (99.999 %, trace metals basis), tetrabutylammonium hydroxide (99.0 %), tetraethylammonium hydroxide (puriss. trace metals basis, 25 % in methanol), H_2_SO_4_ (99.999 %, Sigma–Aldrich), NaClO_4_ (ultra >99.5 %, Fluka), methanol (99.9 %, Uvasol), NH_4_OH (suprapure, 25 % in water), HClO_4_ (suprapure, 70 %, Merck), and ultrapure water (Millipore MilliQ gradient A10 system, 18.2 MΩ^−^ cm, 3 ppb of the total organic carbon). We made every effort to exclude the possibility of impurity deposition during the cathodic polarization. The metal wires used as working and counterelectrodes were provided by Alfa Aesarand Mateck GmbH with purities between 99.98 and 99.999 %, and the Materials Research corporation (very high “MARZ” purity), and the glassy carbon electrodes were provided by HTW Hochtemperatur-Werkstoffe GmbH. The working electrode was mounted on a vertical translation stage which is used to precisely adjusted the submersion depth (measured from the moment the electrode touches the liquid surface) by a micrometer screw.

For the synthesis of the nanoparticles a homemade power amplifier was used (dc >100 kHz, maximum output voltage ±35 V, maximum power 50 W). The dc and ac control signals were generated by a National Instruments DAQ module (NI-6211), which was also used for simultaneous data acquisition.

For the electrochemical measurements of the oxidation of CO and methanol the cell was deoxygenated prior to the experiments by saturating the solution with argon (N66) for 20 min. Argon was also used to deoxygenate all other solutions and for dosing CO (purity N47). The stripping voltammograms of CO were obtained after saturating the cell with CO for 2 min while keeping the Pt electrode immersed in the solution at 0.10 V, followed by argon purging for 20 min to remove excess CO. Finally, the working electrode was brought back into the meniscus configuration and the oxidation of the CO adlayer was followed by scanning the potential from 0.05 to 0.85 V.

All voltammetric experiments were carried out in an electrochemical cell using a three-electrode assembly at room temperature. A platinum wire was used as counterelectrode and a reversible hydrogen electrode (RHE) in the supporting electrolyte was the reference electrode. All potentials are referred to this electrode. The electrochemical measurements were performed with a computer-controlled Autolab PGSTAT12 potentiostat–galvanostat.

The TKK platinum nanoparticles with a diameter of 5 nm on a Vulcan carbon catalyst have been obtained from the group of N. Markovic (Argonne National Laboratory). No effects of shelf-life aging are either known or observed for this industrial catalyst.

## Footnotes

* With the exception of Pd, which we ascribe to the formation of a passivating hydride layer.
