# Supplementary material for: Cathodic Corrosion: A Quick, Clean, and Versatile Method for the Synthesis of Metallic Nanoparticles
Source: Angew Chem Int Ed Engl. 2011 May 27;50(28):6346–50. doi: 10.1002/anie.201100471 (PMC3166651; doi:10.1002/anie.201100471)
Supplement: Supplementary file 1 [file anie0050-6346-SD1.pdf]

Angewandte Chemie
Eine Zeitschrift der Gesellschaft Deutscher Chemiker

Supporting Information
© Wiley-VCH 2011
69451 Weinheim, Germany

Cathodic Corrosion: A Quick, Clean, and Versatile Method for the Synthesis of Metallic Nanoparticles**

*Alexei I. Yanson, Paramaconi Rodriguez, Nuria Garcia-Araez, Rik V. Mom, Frans D. Tichelaar, and Marc T. M. Koper**

anie_201100471_sm_miscellaneous_information.pdf

i. On the possibility of cathodic alloying or formation of intermetallic compounds

ii. On the mechanistic details of metal nanoparticle formation at the cathode-electrolyte interface

iii. On the possibility of physical destruction of the cathode during an extreme cathodic treatment

iv. Figures SI1-SI19

i. **On the possibility of cathodic alloying or formation of intermetallic compounds**

In references [22] and (*S1-S4*), following the pioneering work of Haber [20, 21] and his colleagues (*S5*), it has been proposed that cathodic atomisation under stationary conditions takes place according to the following mechanism. First, the alkali cation is electrochemically reduced and "incorporated" into the cathode, forming an intermetallic compound. Water from the electrolyte then reacts with this intermetallic compound, chemically leaching the alkali and leaving behind the nano-porous metal surface. This mechanism is inconsistent with the following observations:

1. Cathodic corrosion and atomisation is observed not only in solutions with alkali metal cations, but also with ammonium, tetra-(m)etylammonium and tetra-butylammonium cations (Fig. SI9). Those neither alloy nor form intermetallic compounds with metals.
2. At the initial stages of the atomisation process the surface of the electrode shows clear crystallographic etch pits (Fig. SI7), consistent with chemical dissolution. A leached electrode should have a rough spongy texture with strong preference to crystal defects and no preference to crystal planes.
3. Chemical decomposition of the alkali-containing intermetallic compound should be strongly inhibited by going to more cathodic potentials, while the process actually benefits from doing so.

Common ground can be found if we consider that this "intermetallic" phase is the same as what we describe as a "cation-stabilized metal anion". Borrowing on the knowledge from the Zintl compounds we note that in most cases there is not just a single metal anion but rather a polyanion such as Pb_9^{4-} or In_{11}^{8-} stabilized by K^+ or Na^+ cations [31]. Perhaps similar species are created at the interface during cathodic treatment.

S1. A. Frumkin, V. Korshunov, I. Bagozkaya, *Electrochim. Acta* **15**, 289 (1970)
S2. B. N. Kabanov, *Electrochim. Acta* **13**, 19 (1968)
S3. A. I. Chernomorskii, *Soviet Electrochemistry* **13**, 1198 (1977)
S4. L. A. Reznikova, D. P. Aleksandrova, B. N. Kabanov, *Soviet Electrochemistry* **17**, 445 (1981)
S5. M. Sack, *Zeitschrift für anorganische Chemie* **34**, 286 (1903), and references therein

ii. **On the mechanistic details of metal nanoparticle formation at the cathode-electrolyte interface**

As we observe the formation of metal nanoparticles at the cathode-electrolyte interface, the question arises about the nature of this interface at very negative potentials, and consequently, where do nanoparticles form within this interface. Haber already noted that "… no solution remains neutral on the cathode if hydrogen is generated" [21]. In a concentrated aqueous electrolyte at very high reduction current densities the protons at the interface will be depleted and the local pH will therefore be very high. This region, however, will be quite thin and not stationary due to convection caused by vigorous hydrogen gas evolution. The fact that hydrogen keeps evolving at a continuous rate also suggests that "free water", i.e. water molecules not participating in solvation shells, is still able to penetrate this layer and reach the cathode. Therefore describing this interface as a stable one resembling a stationary diffusion layer during typical electrolysis would be oversimplified. While we currently do not have a good model for it, we can say that whatever its true nature is, the metal anions created in it, and the metal nanoparticles they form at more negative (but necessarily oxidizing!) potentials, are existing only in extreme proximity to the electrode. In Fig. SI19 the locality of the process is demonstrated in SEM images, and the frequency dependence in Fig. SI11 shows the existence of an optimum ac frequency range, possibly matching the diffusion coefficient in this interfacial layer. Therefore we must conclude that both during the dc cathodic treatment and during the cathodic half-cycle of the ac treatment, this thin aprotic layer is formed almost instantaneously, and the formation of nanoparticles proceeds in a similar way for both cases. It is due to the other (positive) half-cycle of the ac voltage that the nanoparticles weaken their attachment to the electrode and have a chance at escaping to the bulk solution. This is supported by the fact that for all metals studied (except Pb, Sn and perhaps other "Zintl metals" known to have quite stable anionic complexes) nanoparticles are exclusively found at the surface during dc cathodic polarization, while they readily go into solution under ac treatment.

iii. **On the possibility of physical destruction of the cathode during an extreme cathodic treatment**

The fact that during a -10 V dc cathodic treatment of a 1mm-long piece of 0.1 mm diameter Pt wire the electrochemical current reaches -400mA (Fig. SI3) can be interpreted as dissipation of 10 MW/m^2 at the electrode which would cause boiling of aqueous electrolyte. This, in turn, would cause the transition from “normal electrolysis” to “contact glow discharge electrolysis” (*S6*), causing rapid degradation of the electrode’s surface. To convince the reader that we stay well within the domain of conventional “normal” electrochemistry we note that i) the cathodic treatment at -3 V and -20mA (150 kW/m^2) produces qualitatively the same result albeit in a longer time, and ii) by performing similar cathodic treatment in acid absolutely no changes, let alone damage due to glow discharge, is visible in the SEM micrograms of electrode’s surface (see Fig.SI6). Although it is possible to obtain sparking at the electrode when either using inductive ac source (transformer) or going to extremely high voltages (>30 volts), we can safely exclude this mechanism here based on the abovementioned observations.

S6. U. Gangal, M. Srivastava, S. K. Sen Gupta, *J. Electrochem. Soc.* **156**, F131 (2009)

iv. **Figures**

Fig. SI1

A real-time movie of the process of atomisation of 100um dia. Pt wire in 1M NaOH showing the formation of nano-particles in solution as well as the simultaneous evolution of ac current extrema (black and red) and positive and negative averages (blue and yellow).

Fig. SI2

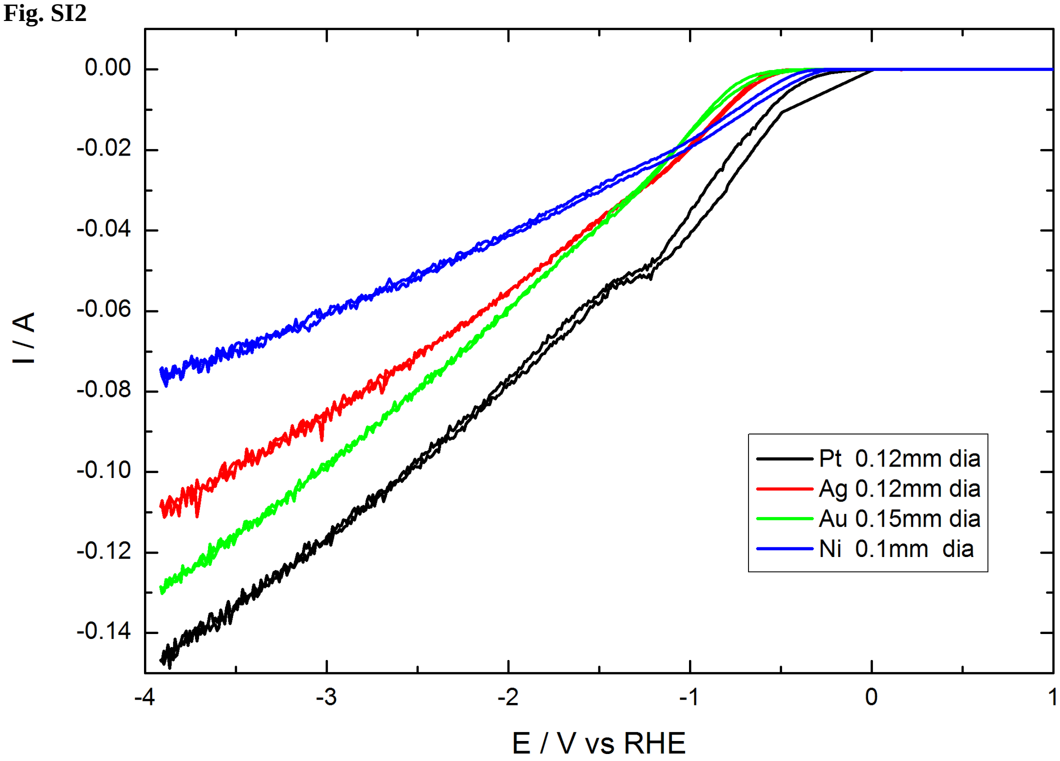

Current-voltage plots of hydrogen evolution reaction on Pt, Ag, Au and Ni wires immersed by 1 mm in 1M NaOH electrolyte, scan rate 50mV/sec. The kink at ~-1V for Pt, which we believe is due to hydrogen bubbles matching the diameter of the wire, is absent for thicker Pt wire.

Fig. SI3

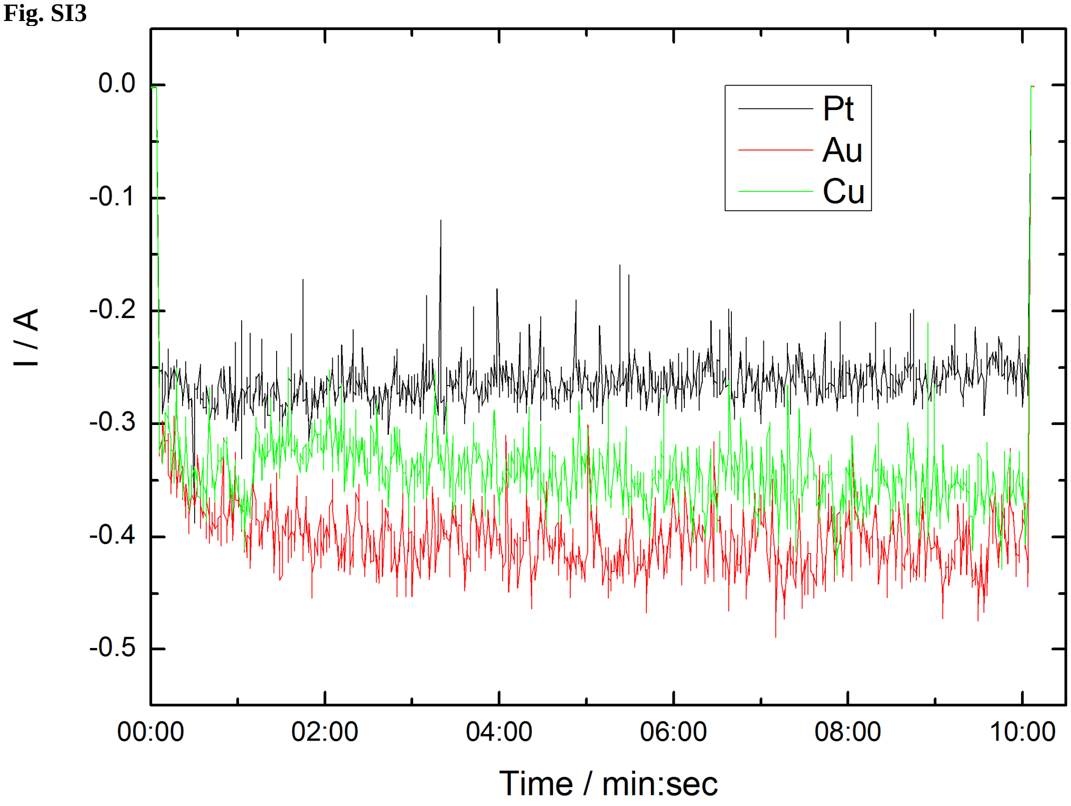

Current-time (chronoamperometry) plots of cathodic polarization at -10 V of Pt, Au and Cu wires immersed by 1 mm in 10M NaOH electrolyte. Apparent noise in the curves is due to hydrogen gas evolution and bubble formation along the wire, causing fluctuations of the electrode surface in contact with the electrolyte.

Fig. SI4

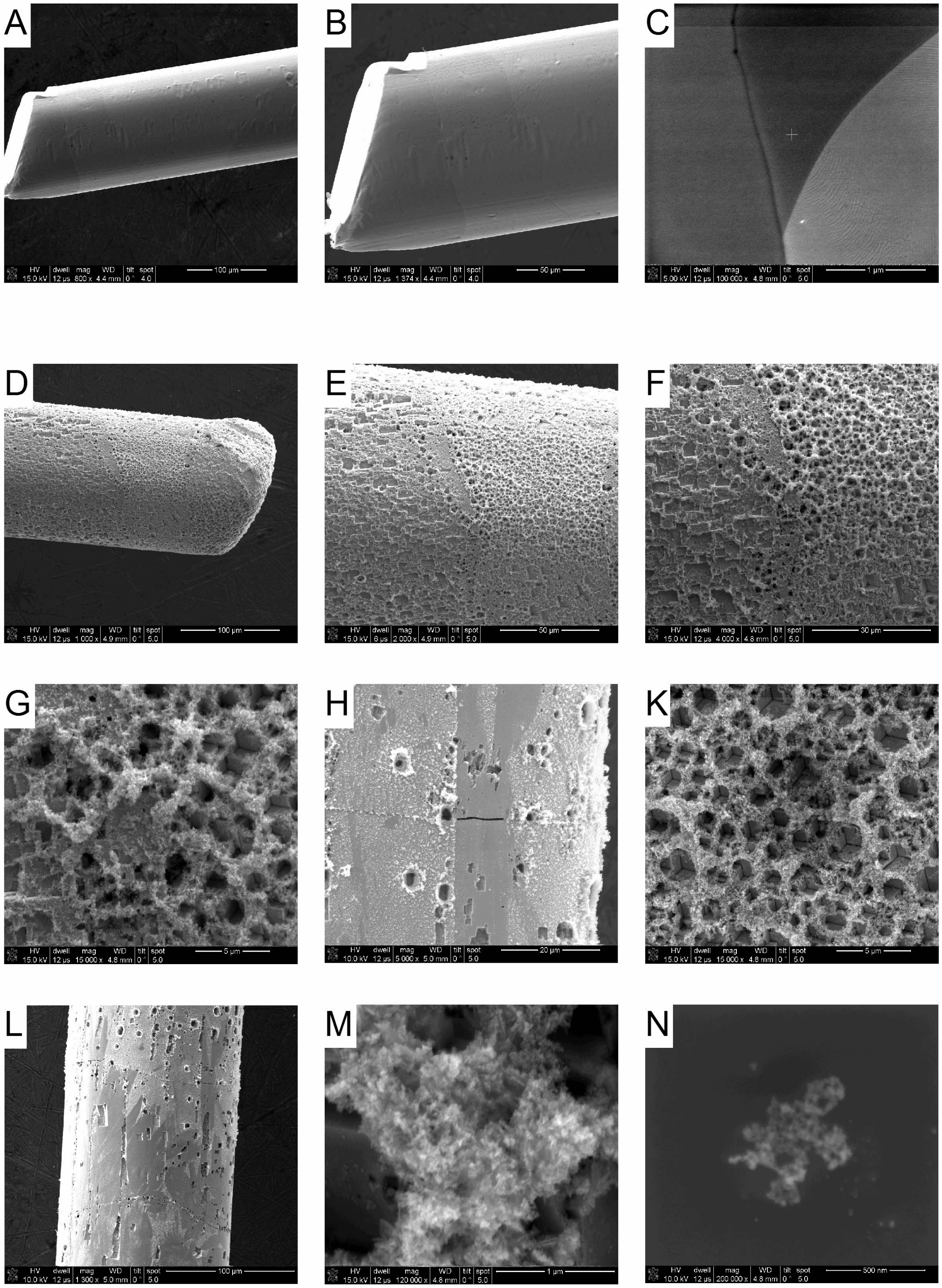

SEM images of the Pt wire used in Fig. SI3. Flame-annealed wire (A-C) shows clear crystalline domains, which reveal different patterns or shapes of etch pits after cathodic treatment (D-N).

Fig. SI5

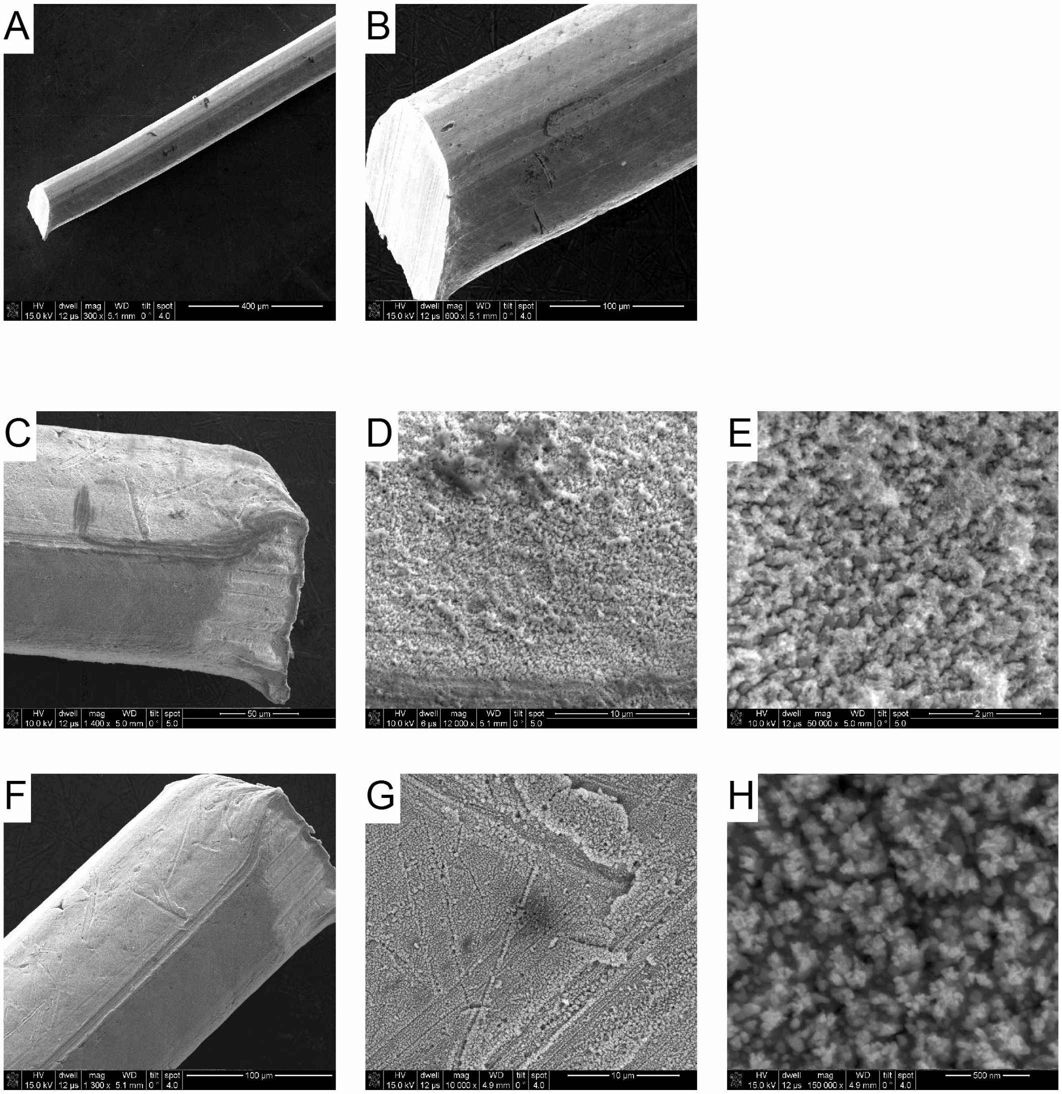

SEM images of the Au wire used in Fig. SI3 before (A, B), and after cathodic treatment (C-H).

Fig. SI6

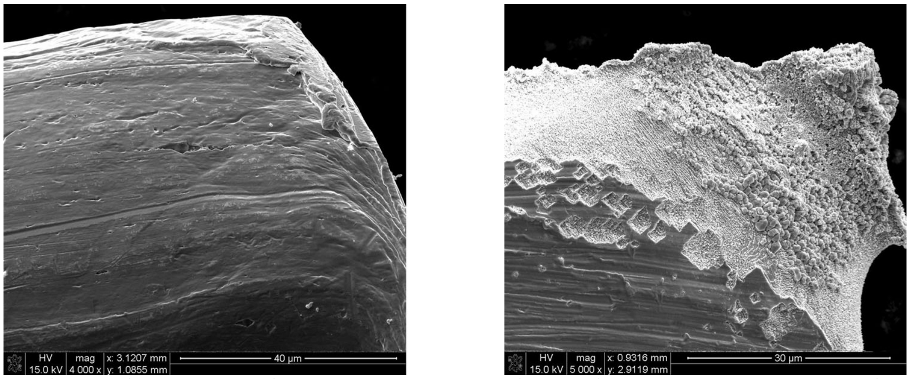

(above) SEM images of a tip of a gold wire after cathodic treatment of -10 V for 30 min in 1M H_2SO_4 (left), and the same treatment in the same solution with 1M Na_2SO_4 added (right). Nanoparticle deposits as well as crystallographic (square) etch pits are visible.

(below) An annealed platinum wire, cathodically treated at -30 V for 20 min in 1M HCl (CE separated) is shown for comparison.

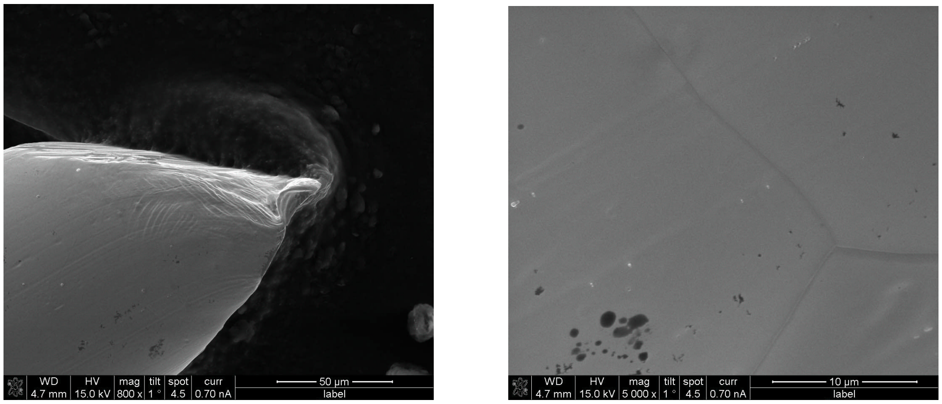

Fig. SI7

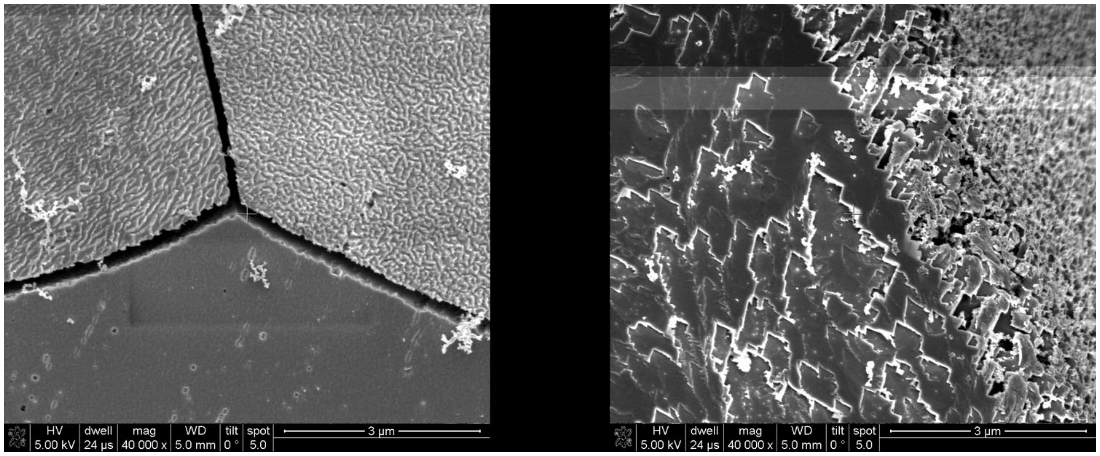

SEM images of annealed platinum wire after cathodic treatment of -4 V for 60 sec in 10M NaOH. Note enhanced etching at the three-grain boundary, chains of nanoparticle deposits (left), and crystallographic etching patterns (right).

Fig. SI8

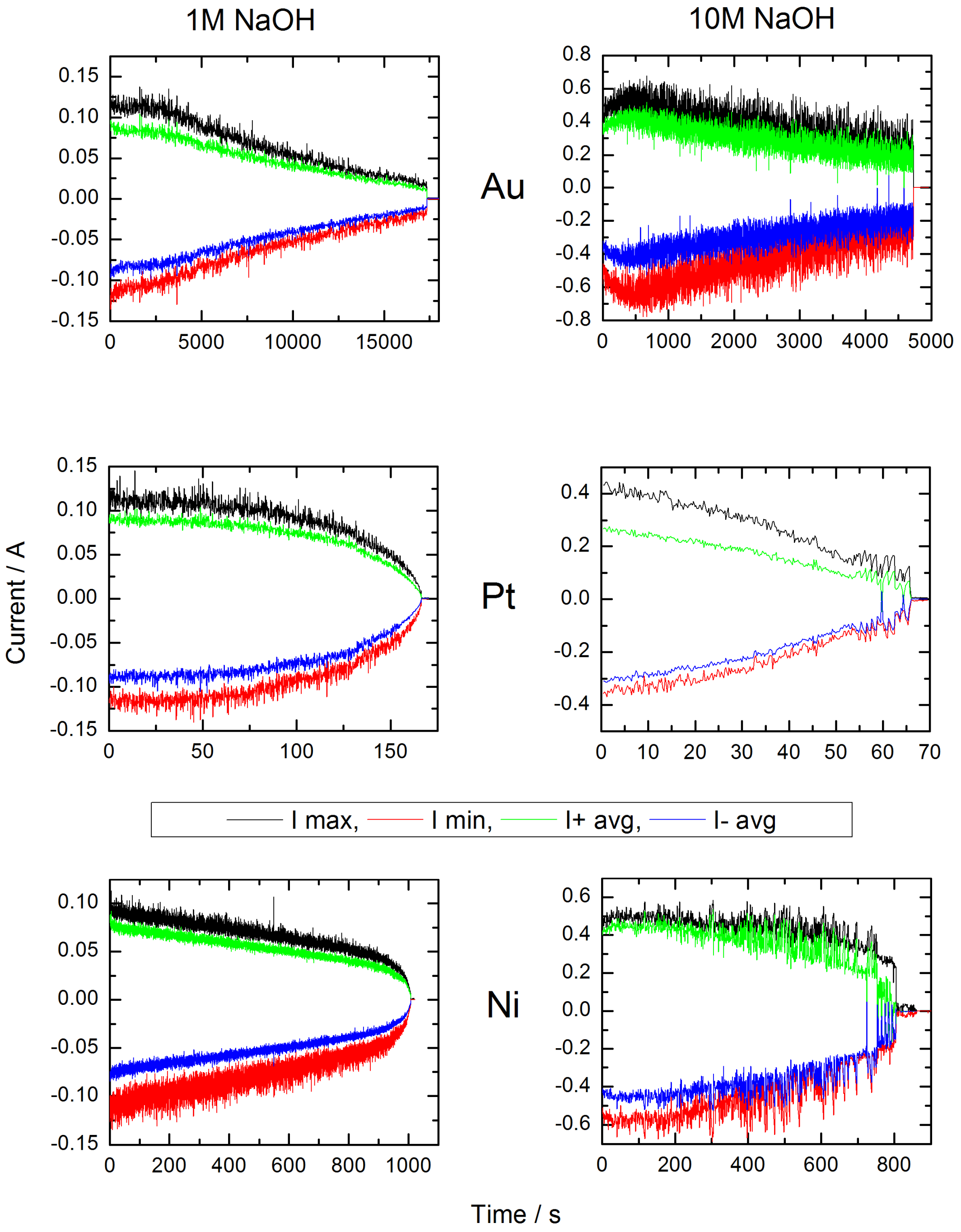

AC current vs. time plots for different metal wires. I max (I min) – absolute maximum (minimum) value of current, I+ avg (I- avg) – average of current measured during the positive (negative) ac half-cycle. Wire thickness varied slightly (0.1 - 0.12 mm dia), but was always submerged by 1mm into the solution. Test solution: 1M and 10M NaOH, as indicated in the figure. 20 Vp-p square wave ac voltage, 100Hz.

Fig. SI9

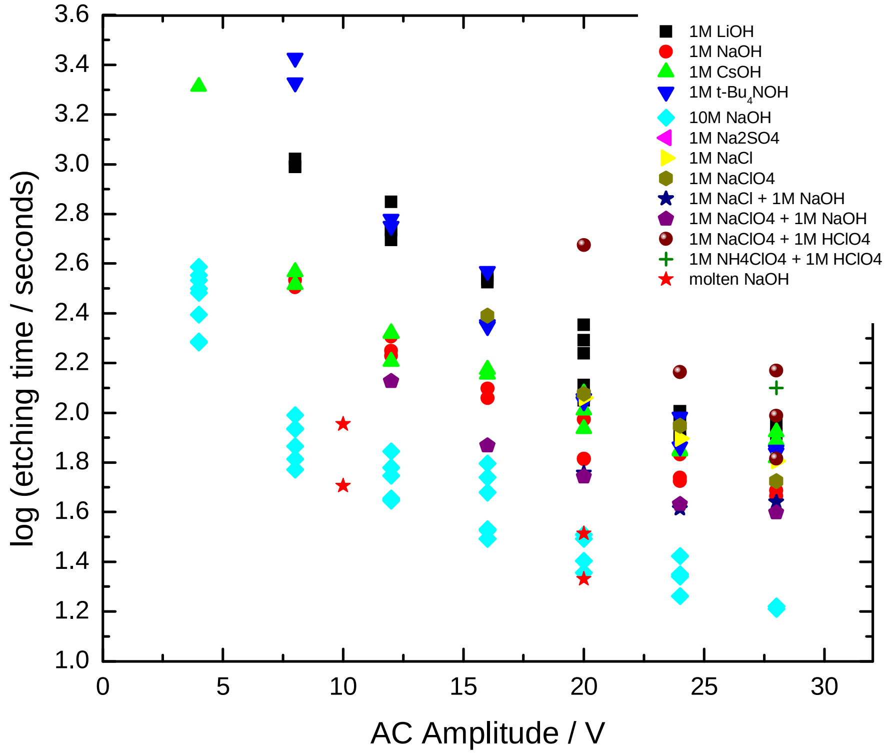

Time required to atomize the 1mm length of 0.125 mm dia. annealed platinum wire as a function of 100 Hz square-wave ac amplitude in various electrolytes.

Fig. SI10

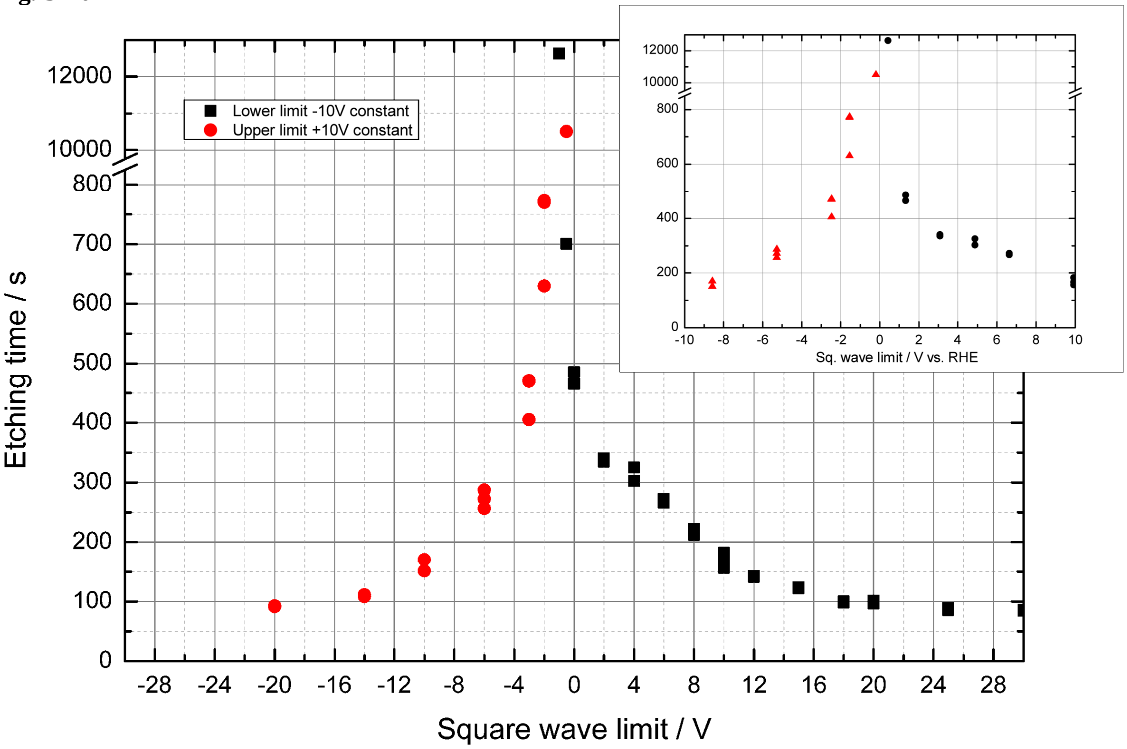

Dependence of atomization time of Pt wire on the limits of the square wave ac voltage applied. Red circles: the upper value of the square wave was fixed at +10 V, and the lower one systematically varied between -20 and -0.5 V. Black squares: the lower value of the square wave was fixed at -10 V, and the upper one varied from -1 to 30 V. For each new value 1 mm of Pt wire was completely atomized, and the time recorded. During these measurements the ac voltage between the Pt wire and a Hg/HgO reference electrode in same electrolyte was recorded and plotted vs. RHE (see inset). Glassy carbon was used as counter-electrode.

Fig. SI11

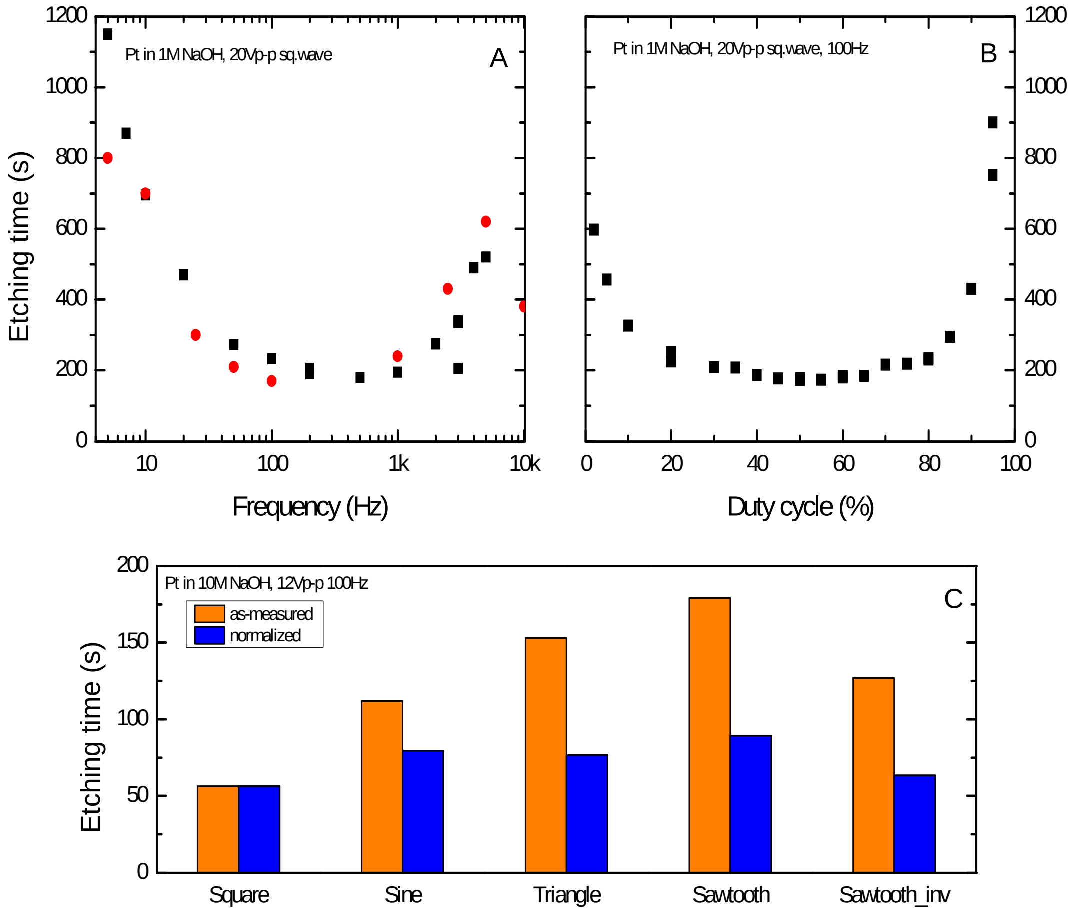

Atomization time dependence on frequency, duty cycle and waveform. Latter is also shown normalized by the area under the waveform. Duty cycle of 20% means the square waveform is positive for 20% of the total oscillation period. Pt electrode wire 0.1mm dia. submerged by 2mm (A) and 1mm (B, C). Black and red points in (A) are from two separate measurement series. Glassy carbon was used as a counter-electrode.

Fig. SI12

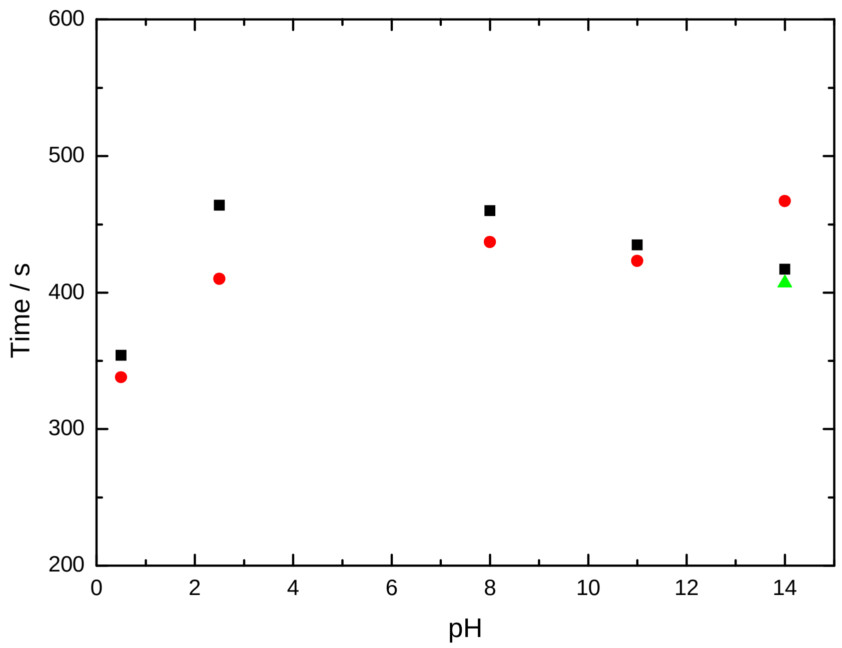

Time required to atomize 0.12 mm dia. Pt wire submerged by 1mm in 0.5M NaOH + x $HClO_4$ solution. 20 V p-p square wave, 100 Hz. Weak (if any) dependence is indicative of the fact that under extreme cathodic (anodic) potentials the pH in the vicinity of the electrode is strongly shifted towards high (low) pH values due to the consumption of protons (hydroxyl ions).

Fig. SI13

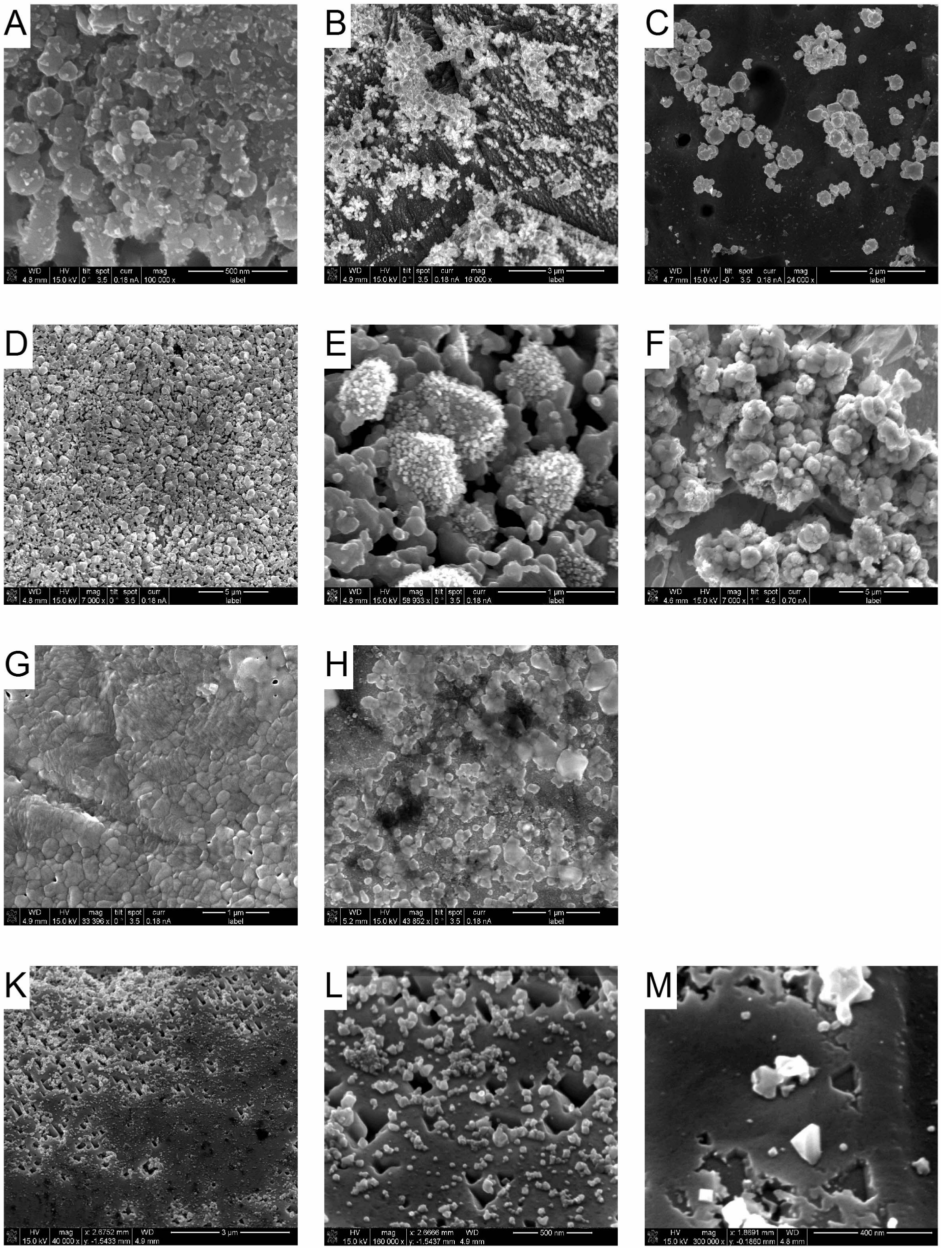

SEM images of cathodically treated metal surfaces. (A) Pt, (B) Au, (C) Rh cathodically treated at -30 V for 20 minutes in 1M NH_4Cl; (D, E) Ag cathodically treated at -30 V for 20 minutes in 1M NaCl; (F) Re cathodically treated at -30 V for 20 minutes in 1M $NaClO_4$; (G, H) Ir cathodically treated at -30 V for 20 minutes in 1M HCl + 1M NaCl; (K-M) Au cathodically polarized in 1M Na_2SO_4 at -10 V for 10 seconds. The counter electrode was placed in a separate compartment to avoid any possible cross-over of (oxidizing) species.

Fig. SI14

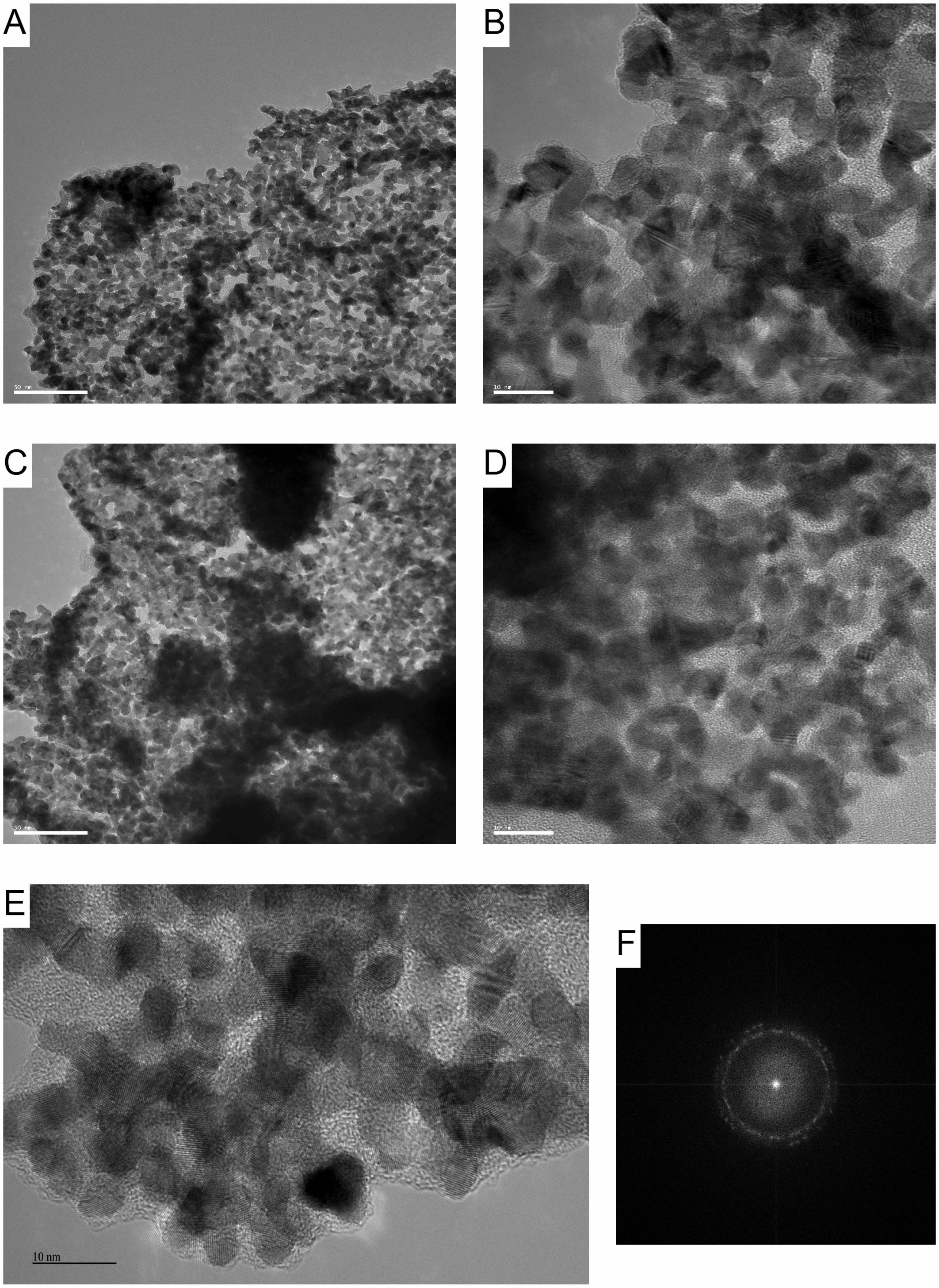

(A-E) TEM of Pt nanoparticles, (F) FFT of (E), showing "diffraction" circles with radii corresponding to lattice spacings of 2.27 Å for {111} and 1.96 Å for {200}

Fig. SI15

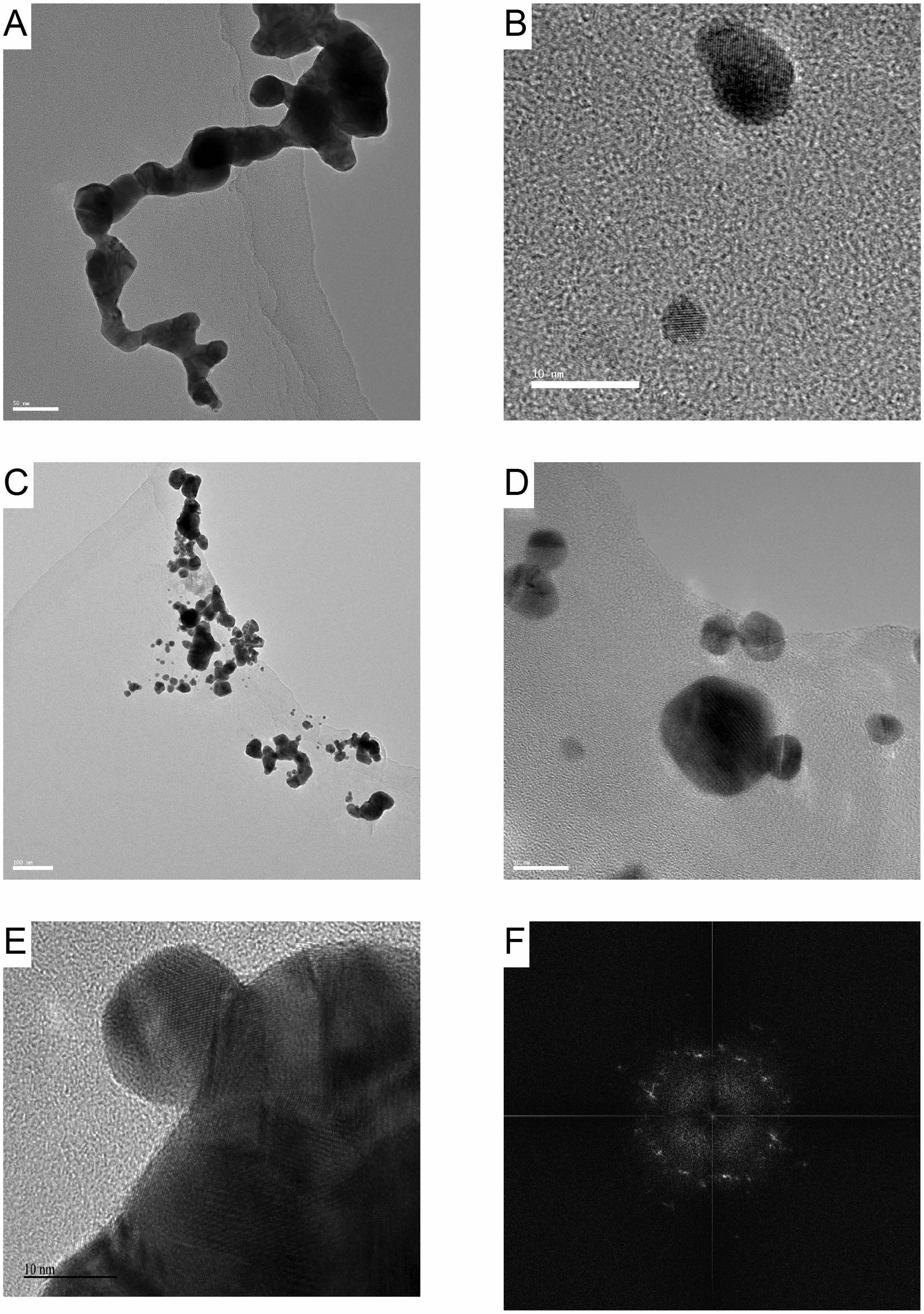

(A-E) TEM of Au nanoparticles, (F) FFT of (E), showing dots on a circle with radius corresponding to Au {111} plane distance of 2.3 Å

Fig. SI16

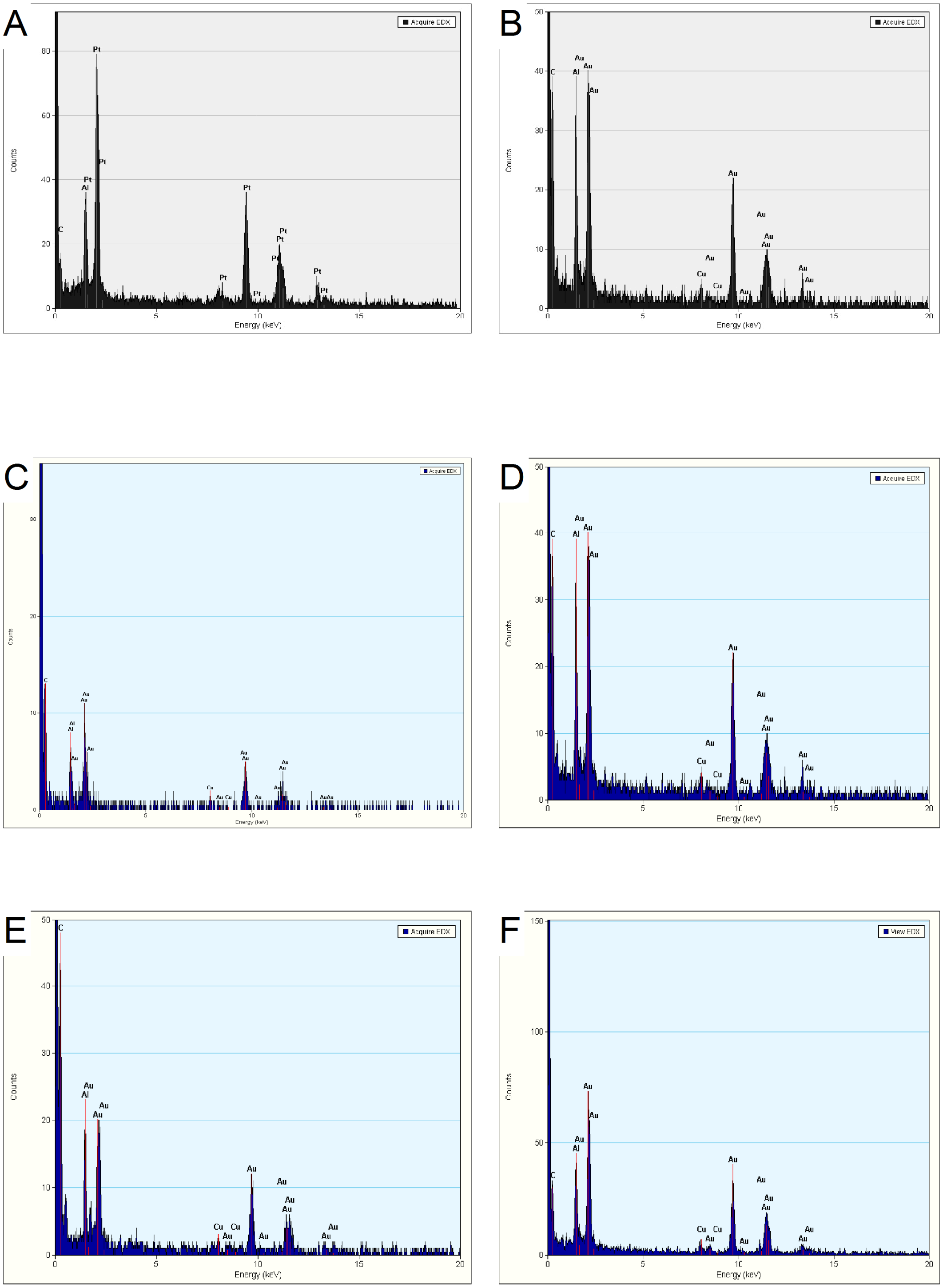

TEM-EDX spectra of Pt (A) and Au (B) nanoparticles. Specifically: (C) - one of the Au nanoparticle in Fig2b of the manuscript; (D, E) - a group and a single NP from panel (C) of Fig.SI15; (F) - the smallest NP in panel (B) of Fig.SI15. Incidental Cu and Al signals originate from the TEM grid and/or holder.

Fig. SI17

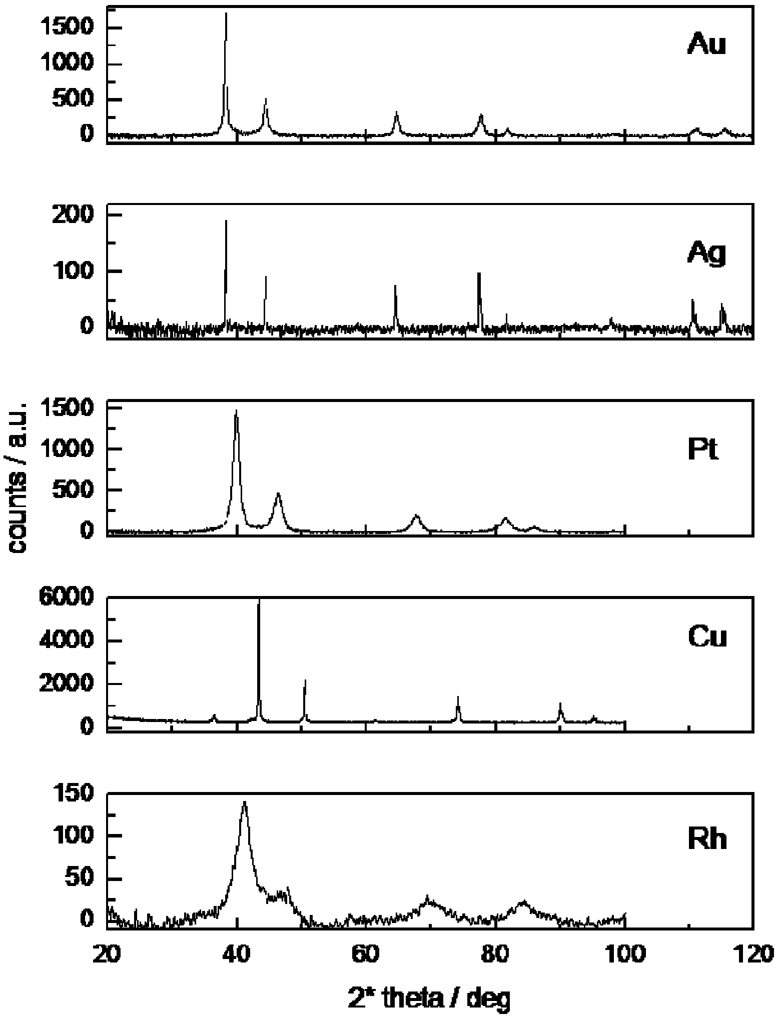

X-ray diffraction spectra of Au, Ag, Pt, Cu and Rh nanoparticles. The positions of the peaks coincide with those expected for the crystal structure of a corresponding metal (in the case of Cu and perhaps Ag some oxide is also visible). From the widths of the peaks we obtain the rough indication for the average crystallite size to be 25, 58, 12, 57 and 6 nm, respectively.

Fig. SI18

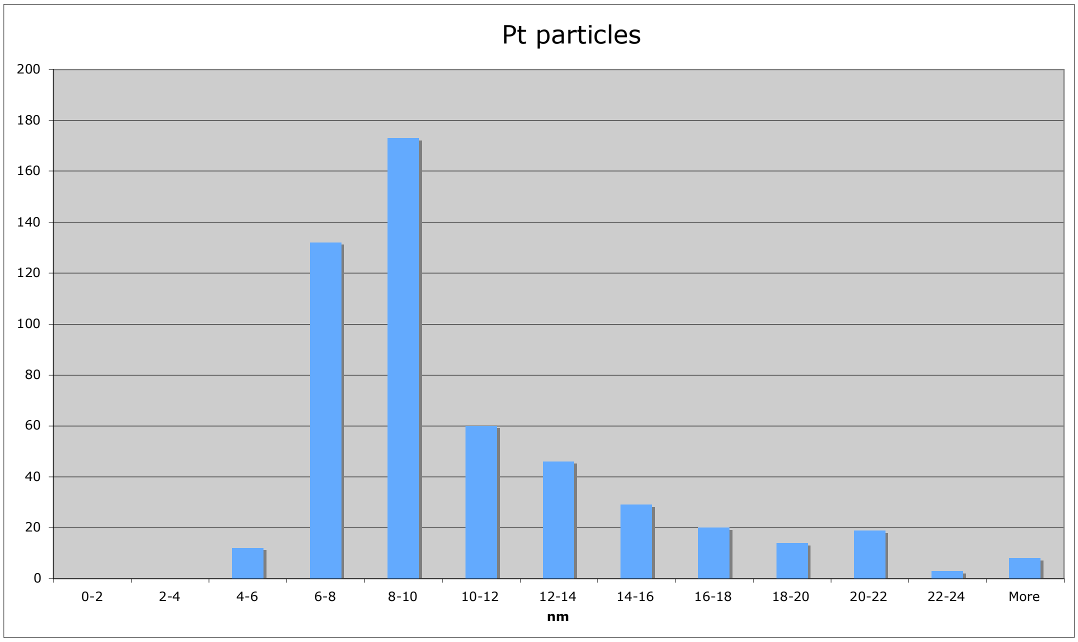
Pt particles
200
180
160
140
120
100
80
60
40
20
0
0-2
2-4
4-6
6-8
8-10
10-12
12-14
14-16
16-18
18-20
20-22
22-24
More
nm

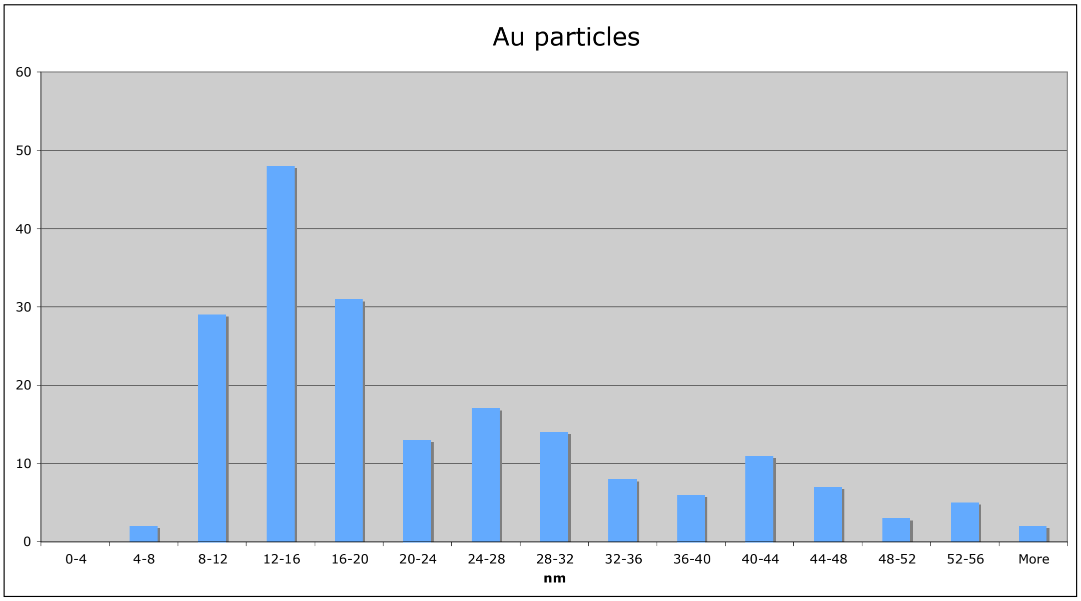
Au particles
60
50
40
30
20
10
0
0-4
4-8
8-12
12-16
16-20
20-24
24-28
28-32
32-36
36-40
40-44
44-48
48-52
52-56
More
nm

Fig. SI19

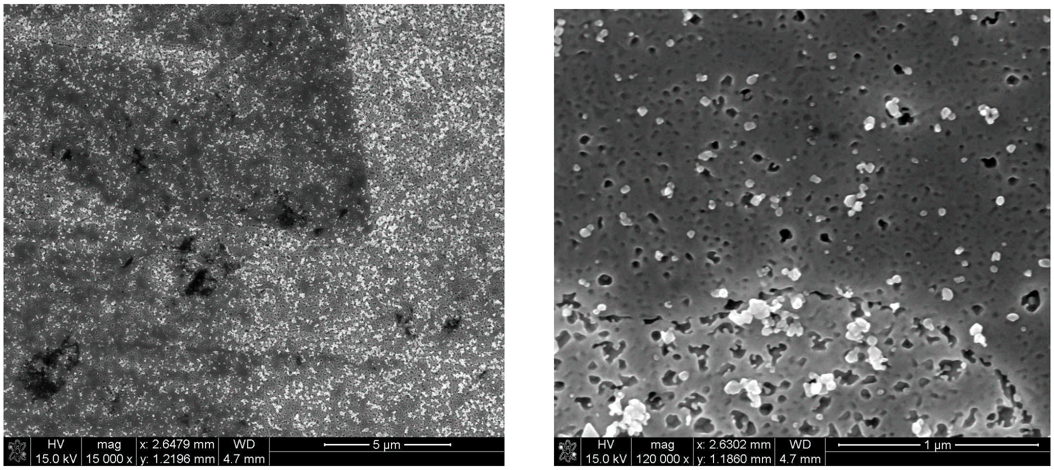

Annealed gold wire cathodically treated at -10 Vdc in an electrolyte containing 1M Na^+ ions. Left image shows a clear crystal grain boundary and different corrosion rates on different crystal orientations. Right image is a zoom-in, showing how within 1 micron of the grain boundary the surface concentration of nanoparticles clearly changes. The latter is an indication of the very limited diffusion of nanoparticle-forming species (metal anions), corroborating our hypothesis of an extremely thin aprotic layer and very short lifetimes of metal anions.
